# Carcinogenic Effects of Areca Nut and Its Metabolites: A Review of the Experimental Evidence

**DOI:** 10.3390/clinpract13020030

**Published:** 2023-02-21

**Authors:** Kalpani Senevirathna, Roshan Pradeep, Yovanthi Anurangi Jayasinghe, Shalindu Malshan Jayawickrama, Rasika Illeperuma, Saman Warnakulasuriya, Ruwan Duminda Jayasinghe

**Affiliations:** 1 Centre for Research in Oral Cancer (CROC), Faculty of Dental Sciences, University of Peradeniya, Peradeniya 20400, Sri Lanka; 2 Faculty of Dentistry, Oral and Craniofacial Sciences, King’s College, London SE1 9RA, UK; 3 Department of Oral Medicine and Periodontology, Faculty of Dental Sciences, University of Peradeniya, Peradeniya 20400, Sri Lanka

**Keywords:** oral cancer, areca nut, carcinogenicity, cytotoxicity, genotoxicity, in vivo and in vitro studies

## Abstract

Oral cancers (OC) are among the most frequent malignancies encountered in Southeast Asia, primarily due to the prevalent habit of betel quid (BQ) and smokeless tobacco use in this region. Areca nut (AN), the primary ingredient in BQ, contains several alkaloids, including arecoline, arecaidine, guvacoline, and guvacine. These have been associated with both the AN abuse liability and carcinogenicity. Additionally, variations in AN alkaloid levels could lead to differences in the addictiveness and carcinogenic potential across various AN-containing products. Recent studies based on animal models and in vitro experiments show cellular and molecular effects induced by AN. These comprise promoting epithelial-mesenchymal transition, autophagy initiation, tissue hypoxia, genotoxicity, cytotoxicity, and cell death. Further, clinical research endorses these undesired harmful effects in humans. Oral submucosal fibrosis, a potentially malignant disease of the oral cavity, is predominantly reported from the geographical areas of the globe where AN is habitually chewed. OC in chronic AN users presents a more aggressive phenotype, such as resistance to anti-cancer drugs. The available evidence on the carcinogenicity of AN based on the findings reported in the recently published experimental studies is discussed in the present review.

## 1. Introduction

Consumption or chewing of areca nut (AN), the seed (endosperm) found in the fruit of the *Areca catechu* tree, is a cultural habit in the tropical countries of South Asia, the Asian Pacific region, and some parts of East Africa [1]. In 2004, AN was classified as a group 1 human carcinogen by the International Agency for Research on Cancer [2]. AN causes oral squamous cell carcinoma (OSCC) and oral potentially malignant disorders (OPMD) such as oral submucous fibrosis (OSF), oral leukoplakia (OL), oral erythroplakia (OE) and lichenoid reactions [1]. In addition, AN-induced OPMDs have recorded various rates of malignant transformation [3,4]. The AN-associated pathogenesis of oral cancer (OC) and OPMDs have been extensively studied during the past few decades through in vitro and in vivo experiments. Previous studies have demonstrated that AN chewers have a significantly higher risk of OC progression [5,6,7,8]. Furthermore, the 5-year survival of a regular AN chewer with OC is lower than that of a never-AN chewer [3,9]. How carcinogenesis is induced in oral keratinocytes by AN and its constituents is well-elucidated, and several mechanisms are described in the literature.

Moreover, among AN chewers, significant increases in the prevalence of cancers in the liver, lung, stomach, pancreas, and larynx were also reported [10]. Several hypotheses on the carcinogenicity of AN are reported in the literature. Thus, the initial pathways of AN associated with carcinogenesis need to be better understood to assess its effects at the molecular level by disentangling the effects brought about by other compounding agents. This review attempts to comprehensively compile the available information, enabling a clear view and understanding of the carcinogenicity of AN.

**Figure 1:**
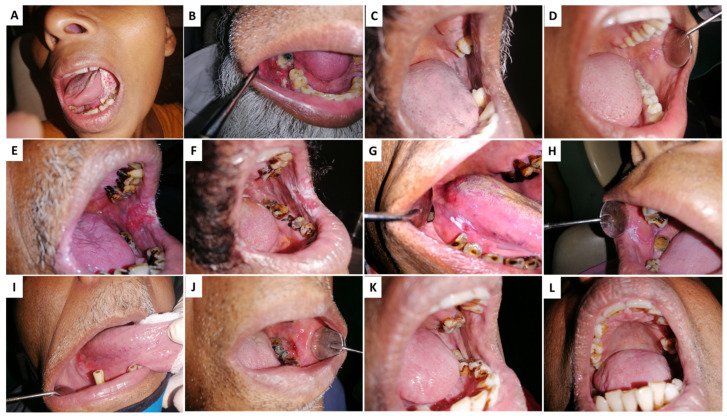
Clinical conditions associated with Areca nut (AN) chewing: (**A**,**B**) Oral squamous cell carcinoma—OSCC, (**C**,**D**) Oral lichenoid reactions—OLL, (**E**,**F**) oral erythroplakia—OE, (**G**,**H**) oral leukoplakia—OL (**I**,**J**) Erythroleukoplakia, (**K**,**L**) Oral lichen planus—OLP.

AN/BQ induces preneoplastic changes in the lining of the oral cavity of its users. Figure 1A illustrates the clinical appearance of rigidifying of the oral mucosa that occurs in a condition referred to as oral submucosal fibrosis (OSF). OSF is characterized by trismus due to reduced fibro-elasticity and inflammation of the oral cavity. In the second clinical manifestation, a bright red velvety patch termed oral erythroplakia is shown (OE) (Figure 1B). More common are whitish plaques or patches on the buccal mucosa or tongue, termed oral leukoplakia (OL) (Figure 1C). Oral lichenoid lesions (Figure 1D) may appear as white, lacy patches. These lesions may cause burning sensation, pain, or discomfort. Lumps or bumps, swellings/thickenings, crusting and/or erosion, and ulceration on the cheeks, tongue, or other areas in the oral cavity characterize oral squamous cell carcinoma (OSCC) (Figure 1E).

## 2. Different Mechanisms of Carcinogenicity

### 2.1. Areca Nut Extract

Research on AN, in the published literature, describes various animal experimental models that have used crude AN extract to simulate the effects of AN chewing. Aqueous extracts, as well as alcoholic extracts of AN, have also been used. As AN is consumed as a whole nut or in other processed forms, it is justifiable to use AN extract in in vitro and in vivo experiments to study its effects. Different concentration gradients of AN extracts have been selected for these studies according to the toxicity assessments in vitro and based on the AN concentration of the saliva in AN chewers [11,12]. Experimental studies conducted during the past few decades, especially using animal models, have demonstrated that AN extract can initiate, promote, and induce OSF, as well as squamous cell carcinomas [13,14] and squamous hyperplasia [15,16]. Moreover, it was reported in several studies that AN extract causes direct genotoxic and cytotoxic effects on the oral mucosa [17,18,19] In Table 1 we summarize the molecular changes observed in in vitro experiments using AN and alkaloids and reported data are described in detail below.

### 2.2. Areca Alkaloids 

The constituents of AN include several alkaloids (0.15–0.67%), polyphenols (11–26%), fats (1.3–17%), saccharides (26–47%), and some crude fiber and tannins such as gallotannic acid, and phiobatannin [6,7,8]. Studies investigating the underlying mechanism of AN-induced carcinogenicity and addictiveness have detected alkaloids, namely arecoline, arecaidine, guvacoline, and guvacine, as the constituents of AN contributing to these actions [2,34,35]. Among the four major alkaloids, arecoline is the primary alkaloid in AN. It regulates a group of cellular enzymes, including matrix metalloproteinases (MMPs) and lysyl oxidase, and inhibits p53 mRNA expression and DNA repair mechanisms [36,37,38]. The contribution of other alkaloids is not well known; however, they can induce alterations in the macromolecules of mammalian cells [37]. In addition, in an AN consumer, these alkaloids undergo nitrosation in the oral cavity to generate AN-derived nitrosamines (N-nitrosoguvacine, 3-methylnitrosaminopropionitrile, and N-nitrosoguvacoline) to damage DNA [39,40,41]. Furthermore, Arecaidine and 7,12-dimethylbenz(a)anthracene (DMBA) interacted synergistically to induce tumorigenesis in the buccal pouch of hamsters [42]. Another study demonstrated that 4-nitroquinoline-1-oxide (4-NQO) and arecoline induce OC in C57BL/6JNarl mice [14,43].

### 2.3. Effect of Areca Nut Extracts on Molecular Carcinogenesis

Studies based on animal models have revealed that AN extract could be an effective tumor initiator/promoter and may provoke potentially malignant lesions in the oral cavity, such as squamous hyperplasia [15,16], OSF [13,14] or cause malignant transformation [16,42,44]. Likewise, in vitro studies reported that AN extract can decline vital dye accumulation (i.e., neutral red uptake), membrane integrity, and cell survival of cultured human buccal epithelial cells (HBEC) dose-dependently. AN extract also leads to DNA single-strand breaks and DNA-protein crosslinking [20,41,45]. Furthermore, different preparations of AN extracts, i.e., acetic acid extract of areca nut (AAEAN), HCl extract of areca nut (HEAN), aqueous extract of areca nut (AEAN), and ethanol extract of areca nut (EEAN) along with arecoline produced cytotoxic and cytostatic effects to varying degrees, and induced variable levels of unscheduled DNA synthesis in Hep2 cells under in vitro conditions in a dose-dependent manner. As mentioned earlier, a potent effect was observed in the most potent properties of arecoline, EEAN, and HEAN [20,21]. Exposure of cultured human oral keratinocytes (HOK) to ripen AN extract significantly reduced population doubling, increased cellular senescence, decreased cell proliferation, and cell cycle arrest at the G1/S phase [22] (Table 1). It was assumed that BQ might promote tumor cell migration by stimulating MMP-8 expression through the MEK/ERK pathway in some upper aerodigestive tract carcinomas. Among BQ ingredients, arecoline is a positive MMP-8 regulator [46]. The effect of prostaglandin endoperoxide synthase (PTGS) on OC development was investigated in terms of exposing AN extract to two human oral cancer cell lines, KB and cellosaurus cell line OEC-M1, and a standard fibroblast cell line (NF) showed that AN extract significantly inhibited cell proliferation in KB, OEC-M1, and NF. Low concentrations of AN extract significantly enhanced the activity of PTGS in OEC-M1 and NF but significantly decreased at high concentrations. Conversely, the activity of PTGS in KB was inhibited considerably by AN extract, and this effect was dose-dependent [47]. 

Moreover, when treated with human oral mucosal fibroblasts, arecoline or AN extract induced an approximately three-fold increase in mRNA levels of the protooncogene *c-jun*, independent of endogenous glutathione (GSH) depletion [48]. Additionally, AN extract, inflorescence of *Piper betle*, AN polyphenol, catechin, and arecoline reduced cell proliferation and survival. In contrast, an aqueous lime extract of BQ was found to increase cell proliferation [49]. Additionally, AEAN induces chromosomal breaks, reduces GSH levels, and delays cell kinetics in mouse bone marrow cells by inducing sister chromatid exchanges likely associated with TP53-dependent changes in cell proliferation [46]. Ethyl acetate and n-butanol extracts of AN and betel leaves have been reported to induce chromosome breaks in human lymphocytes and Chinese Hamster Ovary (CHO) cells [37].

All components of BQ individually enhance chromatid breaks and exchanges in human cells in vitro by a range of 12–37%. In addition, AEAN induced DNA cleavage and enhanced cell proliferation in mouse kidney T1 cells in vitro [20]. Exposure of CHO-K1 cells to AN extract results in increased micronucleus frequency, G2/M arrest, accumulation of hyperploid or aneuploid cells, and cytokinesis failure. These events correlate with the increased disassembly of actin filaments and intracellular H_2_O_2_ levels [23] (Table 1). AN extracts also induce actin reorganization, resulting in morphological changes in fibroblastoid, lamellipodia formation, stress fiber formation in cultured HOK cells, and loss of subcortical actin [50].

Arecoline has also been reported to inhibit cell spreading, migration, and attachment in cultured human gingival fibroblasts in a dose-dependent manner under in vitro conditions [51]. Depletion of glutathione S-transferase (GST) activity and GSH has been manifested in fibroblasts treated with arecoline and cultured HOK [2]. Arecoline exhibited cytotoxicity to human oral fibroblasts in a dose-dependent manner, whereas cellular GST activity was dose-dependently downregulated, thereby preventing increased lipid peroxidation. The addition of extracellular nicotine acts synergistically on arecoline-induced cytotoxicity, showing that arecoline can render human OMF more susceptible to other reactive substances in cigarettes through the reduction of GST. These observations may explain why patients practicing combined tobacco smoking and BQ chewing habits have a higher risk of developing OC [52]. In addition, arecoline inhibits the growth of human KB epithelial cells in a time- and dose-dependent manner by resulting in cell cycle arrest in G2/M and late S phases due to the initiation of Wee 1, phosphorylated cdc2 proteins, cyclin Bl, and inhibition of p21 protein expression in KB cancer cells. However, the effects of arecoline appear to be mediated differently in human gingival keratinocytes. In this case, arecoline stimulated p21 but restricted cyclin B1 and cdc2 proteins. This clarifies that differential regulation of G2/M and S cell cycle-associated proteins in KB and HGK cells plays a vital role at different stages of carcinogenesis [53]. Furthermore, arecoline can stimulate the phosphorylation of H2A histone family member X (c-H2AX), a sensitive DNA damage marker, in HEP-2, 293 cells, and KB, suggesting that arecoline induces DNA damage. Likewise, p53-activated DNA repair and the expression of p53-regulated p21 (WAF1) were suppressed by arecoline [19].

In addition, due to the inhibition of mitochondrial activity and depletion of intracellular thiols of HGF cells, arecoline appeared cytotoxic. Apart from that, arecoline-induced cell cycle arrest at the G2/M phase in a dose-dependent manner in HGF cells in in vitro conditions [17]. HGF exposed to arecoline revealed that four genes related to the maintenance of genome stability and DNA repair were repressed, including *CHAF1*, *CHAF2*, *FANCG/XRCC9*, and *BRCA1* [24] (Table 1). Among them, at the minimum, *BRCA1* response was dose-dependent. Cyclooxygenase-2 (COX-2) and *PTGS2*, involved in cancer initiation and progression, were upregulated in HGF cells. The two proteins, DNAAJA1 and HSP4A1, were also upregulated dose-dependently by arecoline [24]. It has been reported that treating normal oral fibroblasts with AN extract altered the miRNA expression profile. Furthermore, AN extract-induced upregulation of microRNA-23a (miR-23a) was deemed correlated with an elevation of c-H2AX. A correlation between the AN chewing habit and miR-23a overexpression has also been reported in OC patients. Hence, AN-induced miR-23a was associated with a reduced DNA double-strand break repair and *FANCG* expression, which might lead to AN-associated malignancies in humans [54]. 

Furthermore, oral fibroblasts treated with subtoxic AN extract exhibited MMP-2 activation and growth arrest. The supernatant of arrested oral fibroblasts activated the Ak strain transforming (AKT) signaling pathway in OC cells. Subcutaneous co-injection of arrested oral fibroblasts into nude mice significantly increased the tumorigenicity of xenographic oral carcinoma cells. Therefore, this study concluded that AN extract might damage oral fibroblasts and regulate the progression of oral epithelial carcinogenesis via secreted molecules [55]. Several studies have demonstrated the mutagenicity of AN and its components. For example, arecoline N-oxide, the major metabolite of arecoline, was found to be moderately mutagenic in *Salmonella typhimurium* test strains TA 98 and TA 100. 

However, N-acetylcysteine, cysteine, and glutathione could potently inhibit this mutagenicity [56]. Furthermore, an aqueous extract of tobacco-free AN induced mutations in *S. typhimurium* but not in V79 Chinese hamster cells. Conversely, AEAN induced mutations in *S. typhimurium* and V79 Chinese hamster cells and induced gene conversion in *Saccharomyces cerevisiae* and chromosomal breaks in CHO cells. It has also been reported that the AN tannin fraction induced gene conversion in *S. cerevisiae* [36]. An Ames test of *S. typhimurium* strain TA 1535 revealed that AEAN, HEAN, and arecoline were weak mutagens. In contrast, EEAN and AAEAN were strong mutagens, suggesting that the mutagenic potential of arecoline could be significantly increased by other components of AN [57,58,59]. It has also been reported that exposure to AN extract induces mutations at the hypoxanthine phosphoribosyltransferase locus in human keratinocytes, enhancing the intracellular levels of reactive oxygen species (ROS) and 8-hydroxyguanosine. It also affects the frequency of the appearance of micronuclei in the cells, indicating that stress induced by long-term exposure to AN extract increases genetic damage and oxidative stress in human keratinocytes [25] (Table 1).

### 2.4. Areca Nuts-Related ROS Production and Inflammation

ROS, including hydrogen peroxide (H_2_O_2_), hydroxyl radical (HO•), and superoxide anion (O_2_^−^), are composed of O_2_ radicals and non-radical species generated by partial reduction of O_2_. Mitochondrial oxidative phosphorylation primarily mediates the endogenous generation of cellular ROS. However, they can also be produced through interaction with exogenous sources such as xenobiotic compounds. Oxidative stress occurs intracellularly when ROS overwhelm the cellular antioxidant defense system, either through elevated ROS levels or reduced cellular antioxidant capacity [60]. Oxidative stress causes direct or indirect damage to proteins, lipids, and nucleic acids via ROS and is associated with carcinogenesis [61], atherosclerosis, diabetes [62], neurodegeneration [63,64], and aging [65]. However, the involvement of ROS in the pathogenesis of disease states is not limited to macromolecular damage. It is becoming increasingly evident that ROS signaling contributes to disease development. For example, ROS has been reported to promote tumor metastasis through gene activation [66]. As already mentioned, AN, in combination with lime, forms ROS such as HO• [39,67]. The formation of HO• is promoted by the auto-oxidation of polyphenols from AN by Haber–Weiss or Fenton reactions in the presence of transition metals [68]. 

Mechanisms include enhancement of ROS production by mitochondrial metabolic enzymes such as cytochrome P450s (CYPs) [69], NADPH oxidase enzymes NOX-4 and NOX-1 [26] (Table 1), and the inhibition of the antioxidant system by suppressing superoxide dismutase activity [70,71]. Additionally, arecoline has been reported to induce ROS production in several cell types. For example, in endothelial cells, it stimulates ROS production to suppress the expression of the cytoprotective enzyme hemeoxygenase-1 (HO-1) [72]. HO-1 is a stress protein that regulates a cytoprotective response to diminishing cellular damage [73]. Furthermore, AN elevates the expression of the Interleukins-1b (IL-1b), IL-6, IL-8, and tumor necrosis factor-a (TNF-a), in human peripheral blood mononuclear cells (PBMC) [74,75] and lipid mediators’ leukotriene B4 and prostaglandin E_2_ (PGE2) in neutrophils [76,77]. 

**Figure 2:**
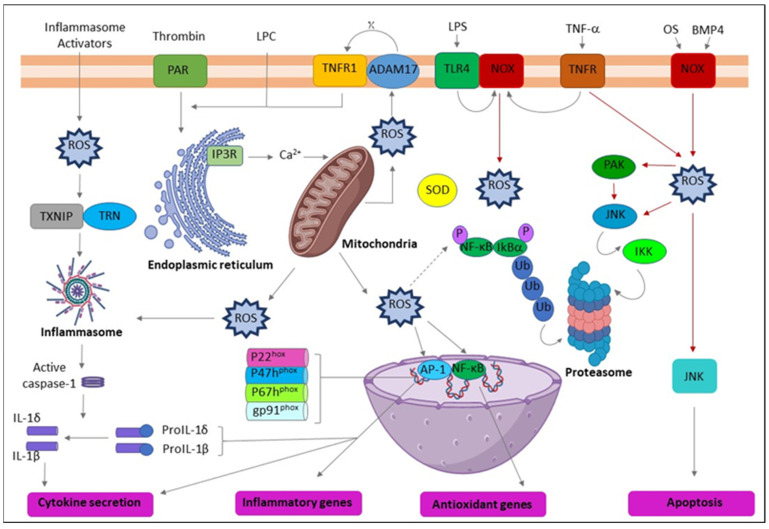
Schematic representation of the action of reactive oxygen species (ROS) leading to inflammation. ADAM17 (ADAM metallopeptidase domain 17); ASC (Activating signal co-integrator 1); BMP4 (Bone morphogenetic protein 4); IKB-α (Inhibitor of nuclear factor kappa B kinase regulatory subunit alpha); IKK (Inhibitor of nuclear factor kappa-B kinase); IP3R (Inositol 1,4,5-trisphosphate receptor type 3); JNK (c-Jun N-terminal kinase); LPC (Lysophosphatidylcholine); LPS (Lipopolysaccharide); NF-κB (Nuclear factor kappa subunit B); NLRP3 (NLR family pyrin domain containing 3); NOX (NADPH oxidase); OxPL (Oxidized phospholipids); PAR (Par family cell polarity regulator); PAK (p21 (RAC1) activated kinase); SOD (Superoxide dismutase); TLR4 (Toll-like receptor 4); TNF-α (Tumor necrosis factor alpha); TNFR (TNF receptor superfamily); TNFR1 (TNF receptor superfamily 1); TXNIP (Thioredoxin interacting protein); Ub (Ubiquitin).

Clinical research has reported the elevated expression of various proinflammatory cytokines by PBMCs in OSF patients [78] and several inflammatory mediators in OSF tissues [79] and OC patients [80,81]. The evidence suggests that regular exposure to AN by habitual chewers may lead to the long-term expression of myriad proinflammatory mediators by immune cells and create a proinflammatory oral microenvironment with the potential for cancer development [82]. PGE2, IL-1α, and COX-2 are inflammatory mediators commonly identified in various tumorigenesis, including OSCC [80,83,84]. Blocking the expression of IL-1α or COX can reduce tumor development [85]. Conversely, increased production of PGE2 may allow malignant clones to evade immune detection [84]. Interestingly, IL-1 is also a potent stimulator of the upregulation of PGE2, COX-2, and other cytokines [86,87]. Correspondingly, AN extract or arecoline induces the generation of ROS in keratinocytes and fibroblasts; following transforming growth factor (TGF-β), IL-6, extracellular signal-regulated kinase (ERK), Ras, and epidermis growth factor receptor (EGFR) are stimulated [26,27,28] (Table 1). Various cytokines or signaling pathways in response to AN treatment could be cell-type specific. Clinically, adjacent tissues of OSF and OC patients from a prevalent chewing area have increased inflammation-related cells [88]. Briefly, AN increases ROS levels, enabling cellular inflammation and tumor progression through multiple molecular regulators (Figure 2). 

### 2.5. AN-Induced Cell Motility and Epithelial-Mesenchymal Transition (EMT)

Cell motility is a crucial characteristic of the malignancy reaction for most cancer invasions and metastasis. MMP and tissue inhibitors of metalloproteinase (TIMP) are essential factors in OSF [89] and OC [90]. The definite mechanism for the malignant transformation of healthy oral epithelium remains ambiguous. MMPs play a crucial role in extracellular matrix (ECM) degradation, a process essential for tumor growth, invasion, and metastasis. The MMP family comprises ≥ 28 members classified as collagenases, gelatinases, stromelysins, matrilysins, or membrane-type MMPs, primarily based on substrate specificity and their sequence homology [91,92].

The involvement of the gelatinases, such as MMP-2 and MMP-9, with the development and progression of cancer is well documented [91,92]. TIMPs control the enzymatic activity of MMPs. TIMPs counteract MMP’s enzymatic activity. Four varieties had been identified, comprising TIMP-1, 2, 3, and 4. TIMP-1 and TIMP-2 can inhibit all non-membrane-kind MMPs, which include MMP-9 and MMP-2. The elevated MMP levels and the decreased levels of inhibitors may enable tumor progression and development [91,92]. Numerous molecular signaling pathways, including p38, mitogen-activated protein kinase (MAPK), Erk1/2, NF-kB, and phosphoinositide 3-kinases (PI3K), could be involved in the modulation of TIMP and MMP expression [91,92,93]. These can act via the muscarinic M4 receptor [93]. According to the clinical findings, excessive MMP-1 or MMP-9 are detected in the cancer tissues or saliva specimens of OC patients who consumed AN [93,94,95]. The AN induces cell motility via the activation of MMP. However, various MMP proteins may respond specifically in different individuals. 

EMT, critical for proper development during embryogenesis and wound healing, is involved in several pathological processes, including degenerative fibrosis and cancer [96,97,98,99]. Although this process was initially described as an “epithelial-to-mesenchymal transformation”, this trans-differentiation process is now termed EMT to emphasize the transient nature of the transformation of epithelial cells into motile mesenchymal cells [97]. Various molecular processes are activated during EMT, including transcription factor activation, epithelial cell surface protein downregulation, loss of connectivity, and apical-basal polarity by epithelial cells. In addition, EMT is associated with the reorganization and expression of cytoskeletal proteins, upregulation of mesenchymal markers, formation of ECM degrading enzymes, reprogramming of gene expression by specific microRNAs, and changes in cell shape from cuboidal to fibroblastoid [97,98,99].

Eventually, all these processes elevate the motility of individual cells and allow the development of an invasive phenotypic feature capable of degrading the basement membrane and migrating through the ECM to colonize numerous territories during embryonic development and cancer progression [96,100,101,102]. Recent studies have proven that AN extract would stimulate oral fibrogenesis and carcinogenesis through the ECM. AN extract or arecoline induces fibroblast trans-differentiation in buccal mucosa fibroblasts (BMFs), which may drive by EMT-related transcription elements, including Twist, Slug, and Zinc finger E-field binding homeobox 1 (ZEB1) [29,103,104] (Table 1). ZEB1 may participate in the pathogenesis of AN-associated OSF by activating the α-smooth muscle actin (α-SMA) promoter and inducing myofibroblast trans-differentiation from BMFs [103]. Further, the upregulation of Twist might be involved in the pathogenesis of AN-associated OSF through dysregulation of myofibroblast activity [103]. AN induces fibrotic activation preassembly through the induction of the EMT process via TGF-β signaling pathways in epithelial cells or gingival fibroblasts [30,105] (Table 1).

**Figure 3:**
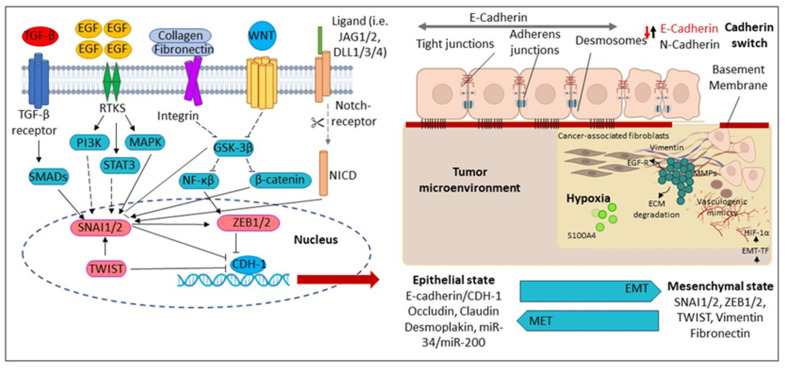
Molecular mechanisms regulating Epithelial-Mesenchymal Transition (EMT). During EMT, the epithelial cells are converted into mesenchymal-like cells. The reverse transition from mesenchymal to epithelial cells is known as a mesenchymal–epithelial transition (MET). CDH1 (Cadherin 1); DLL1/3/4 (Delta-like canonical Notch ligand 1); ECM (Extracellular matrix); EGF (Epidermal growth factor); EGFR (Epidermal growth factor receptor); EMT-TF (Epithelial-mesenchymal transition-transcription factors); GSK3β (Glycogen synthase kinase-3 beta); HIF-1α (Hypoxia-inducible factor-1); *JAG2* (Hs00171432_m1), *JAG1* (Hs01070032_m1); MAPK (Mitogen-activated protein kinase); MMPs (Matrix metalloproteinases); NICD (Notch Intracellular Domain); PI3K (Phosphatidylinositol 3 kinase); SMAD (Suppressor of Mothers against Decapentaplegic); SNAI1 (Zinc finger protein); STAT3 (Signal transducer and activator of transcription 3); TGF-β (Transforming growth factor-β); TWIST1 (Twist-related protein-1); WNT (Wingless/Integrated pathway); ZEB1/2 Zinc finger and homeodomain transcription factor.

Consistently, in either cancer cells or oral keratinocytes, AN mediates the EMT process by decreasing epithelial markers (E-cadherin, involucrin) and increasing mesenchymal markers (N-cadherin, vimentin) via activating the phosphoinositide-3-kinase–protein kinase B/Akt (PI3K-PKB/Akt) pathway [106,107]. Additionally, chronic or long-term AN treatment in cancer or oral epithelial cells promotes mesenchymal trans-differentiation, with the induction of multiple EMT-associated transcription factors; Slug, Twist, ZEB1, Snail, Grp78 and forkhead box C2 (FOXC2) [96,108,109]. Furthermore, Keratin 17 (Krt-17), which belongs to the keratin family, is upregulated upon the AN treatment and facilitates cell motility and malignant transformation via EMT conversion in a mouse model study [15]. In addition, the EMT-associated factor Slug has been upregulated in oral fibroblastic tissues and correlated with different myofibroblast markers, α-SMA [29,103]. Furthermore, loss of E-cadherin expression and elevation of EMT-associated transcription factors or Krt-17 were also significantly associated with OC in habitual BQ chewers [15,94,110] (Figure 3).

### 2.6. Areca Nut Stimulates Autophagy and Restrains Tumor Suppressors

Autophagy is a self-repair mechanism by which cells degrade defective or damaged cellular components and recycle intracellular proteins to ensure survival in hostile environments. Failure of autophagy leads to cell death by either apoptosis or necrosis. Autophagy can play a dual role in carcinogenesis, but in most cases, it promotes tumorigenesis [111]. Cancer cells can upregulate autophagy to withstand microenvironmental stress and increase aggressiveness and growth. Autophagy promotes cancer by suppressing the induction of the tumor suppressor protein p53 and maintaining mitochondrial metabolic function [112]. AN can induce autophagy through clathrin-mediated endocytosis [112]. Additionally, beclin-1, Autophagy related 5 (Atg5), and MEK/ERK pathways are commonly required for AN-induced autophagy. Long-term AN usage might elevate the resistance of survived tumor cells against serum-limited conditions [113,114].

Further, microtubule-associated protein light chain 3-II (LC3-II) transition and Poly (ADP-ribose) polymerase (PARP) cleavage mechanisms were still detected in the serum-starved cells after AN treatment, suggesting simultaneous activation of apoptotic and autophagic pathways [115]. Moreover, p38 activation and MAPK phosphatase (MKP-1) upregulation occurred after AN treatment. AN treatment-induced autophagy in OC cells by the accumulation of LC3-II, formation of autophagosomes, and appearance of enhanced green fluorescent protein-protein light chain 3 (EGFP-LC3) puncta. This induction was mediated through activation of MKP-1, hypoxia-inducible factor-1α (HIF-1α), and p38. Autophagy can be reduced by knocking down AN-modulated HIF-1alpha expression. Furthermore, blocking AN-induced autophagy increased the proportion of OC cells undergoing apoptotic death [116].

In addition to inducing autophagy, AN may inhibit tumor suppressor molecules and induce malignant transformation. Arecoline has been shown to contribute to oral carcinogenesis by inhibiting p53 and DNA repair. Moreover, arecoline induced γ-H2AX phosphorylation, suggesting that DNA damage was mediated by arecoline. This phenomenon was confirmed by the observation of arecoline-induced hyperphosphorylation of Nbs1, ATM, Chk1/2, Cdc25C, and p53 and G2/M cell cycle arrest, suggesting that the cellular DNA damage response was activated. As previously mentioned, arecoline may inhibit p53 through its expression and transactivation functions. As a result, expression of WAF1 and p53-activated DNA repair were suppressed by arecoline [19].

**Figure 4:**
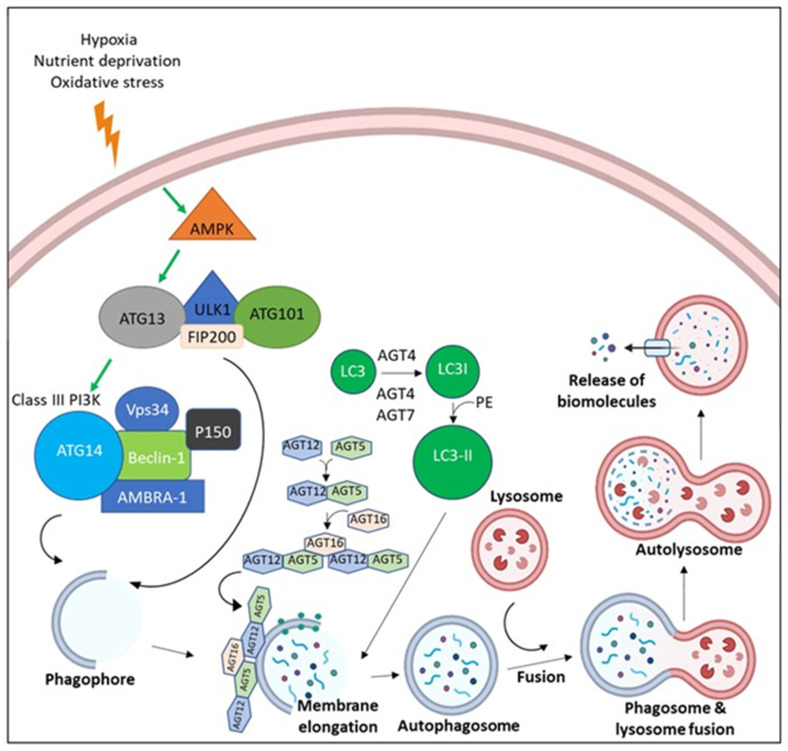
The molecular pathway of Autophagy. Due to the microenvironmental stress, AMP-activated protein kinase (AMPK) is activated, leading to the activation of ULK-1 complex (ULK-1, ATG13, ATG101, and FIP200). ULK-1 complex activation leads the assembly of Class III Phosphoinositide 3-kinases (PI3Ks) (Beclin-1, Vps34, AMBRA, p150, and ATG14). Both ULK-1 complex and Class III PI3K translocate to the nucleation site and stimulate the establishment of the isolation membrane known as the phagophore. Elongation of the phagophore befalls via the effect of ATG5-ATG12-ATG16 and LC3-II until a double membrane vesicle is formed, known as the autophagosome. Autophagosomes fuse with the lysosome, which leads to degradation of cargo via the effect of lysosomal enzymes with release of biomolecules.

Furthermore, p21 and p27 levels were elevated in two OSCC cell lines with high confluence [117]. In addition, elevated levels of p21 and p27 may be downregulated by the ROS/mTOR complex 1 pathway upon treatment with arecoline. Arecoline also leads to ROS-induced DNA damage. This suggests that reduced levels of p21 and p27 may promote the G1/S transition of the cell cycle, resulting in error-prone DNA replication [117]. Briefly, AN may contribute to cellular transformation by activating cellular stress response mechanisms; ROS generation, autophagy induction, and tumor suppressor inhibition (Figure 4).

### 2.7. Areca Nut Consumption Evokes Genotoxicity, Cytotoxicity, Cell Cycle Arresting, and Apoptosis

AN extracts exert cytotoxic and genotoxic effects on HBEC^27^, possibly related to its ability to elevate DNA strand breaks, micronucleus formation, gene mutation, and promote chromosomal abnormalities [118,119]. Arecoline is reported to induce a genotoxic effect [118,119,120]. Additionally, it has been revealed that arecoline can induce cell cycle arrest at the G2/M stage [17,53], which is a consequence and a characteristic feature of cells with DNA damage. In a mouse model study, AN alkaloids have induced sister chromatid exchanges. Arecoline, the principal alkaloid of AN, is clastogenic in many studies [118,119,120]. Arecaidine, another alkaloid, is also reported to be genotoxic in sister chromatid exchange induction assays [31]. In a transgenic mouse study, the frequency of mutations at G:C sites, where G:C→T:A transversions were most frequent, followed by G:C→A:T transitions and G:C→C:G transversions were increased in arecoline, suggesting that arecoline poses a mutagenic hazard in the oral tissues of transgenic mice [32] (Table 1). 

Further, arecoline may prompt cytotoxicity in oral mucosal epithelial cells and fibroblasts; nevertheless, its underlying mechanisms are not fully understood [27,49,121]. Cell cycle arrest, PGE2 synthesis, and cytotoxicity to primary oral keratinocytes and KB cancer cells are some of the cellular and biochemical processes induced due to AN ingredients [27]. Preexisting literature has reported that exposure of human KB cancer epithelial cells, CHO-K1 cells, and oral mucosa fibroblasts to arecoline can evoke G2/M cell cycle arrest and even apoptosis [23,52,53]. Exposure to >0.2 mM arecoline reduces the proportion of EAHY cells that exist in the S phase [122]; however, it increases the cell arresting in the G2/M phase, suggesting that the anti-proliferative and cytotoxic effects of arecoline are feasibly correlated with the changes of cell cycle regulatory proteins such as checkpoint kinases, ATM, p53, and cdc25C for G2/M checkpoint [19,121]. Still, arecoline-induced DNA damage, p21 and p53 expression, and G0/G1 arrest in cultured rat hepatocytes [33] (Table 1). This indicates that the cell cycle response to arecoline differs amongst cells, possibly due to distinct cellular metabolic enzymes in different tissues. Furthermore, arecoline’s prolonged cell cycle dysregulation may result in chromosomal aberration, aneuploidy, and genomic instability [123,124]. 

AN and arecoline induce G2/M cell cycle arrest of oral epithelial cells via triggering Chk1/Chk2 signaling pathways to offer the time for DNA repair [17,82] because AN and arecoline are known to exhibit genotoxicity [125]. In oral keratinocytes, AN may cause cellular senescence and cell cycle arrest through the upregulation of p21, p38, p16, NF-B, COX-2, and IL-6 [22]. In addition, Ras may regulate p53, further affect cdc25 and cyclinB1, and induce cellular senescence [126], signifying Ras activation in the modulation of oral carcinogenesis. As previously indicated, the primary alkaloid of AN, arecoline, is known to cause ROS generation and apoptosis. In addition, arecoline may inhibit AMP-activated protein kinase (AMPK) through intracellular ROS, which is responsible for the execution of apoptosis [116]. Briefly, different molecular pathways and cytotoxic effects in response to AN stimulation may depend on differential microenvironmental factors or specific cell types. Generally, growth arrest or apoptosis is considered the optimal cellular defense mechanism to evade further effects of malignant transformation.

### 2.8. Areca Nut Promotes Malignant Transformation by Inducing Tissue Hypoxia

Hypoxia in the tissue microenvironment alters cellular metabolism and induces various pathological reactions [127]. These hypoxic conditions may be correlated with cellular oxidative stress [69,127]. Additionally, it activates the anaerobic respiratory pathway by increasing the enzymes lactate dehydrogenase, glucose transporter, or hypoxia-inducing factor (HIF) [128,129,130]. It is recognized that hypoxia is a crucial underlying element in the development of tumors and cancers. Several cellular mechanisms, namely sustained HIF proliferative signaling, dysregulated metabolism, and angiogenesis [131,132], are reported to be influenced by hypoxia and HIF signaling. Hypoxia plays a critical role in these hallmarks [133]. HIF-1 is a crucial mediator of cellular adaptation to low O_2_ levels. HIF-1 is a heterodimer comprised of α and β subunits. HIF-1α is an O_2_-regulated subunit induced in response to various stimuli, such as cytokines, growth factors, and hypoxia [134]. 

It has been suggested that the etiology of OSF related to AN chewing may be influenced by hypoxia through the expression of HIF-1 [135]. OSF intrinsically expresses elevated levels of HIF-1α protein than BMF, signifying the presence of the localized hypoxic condition in OSF tissues [136]. Arecoline also could promote HIF-1 expression in BMFs. By stimulating the plasminogen activator inhibitor (PAI-1), which promotes the deposition of ECM in the oral submucosa, hypoxia through HIF-1 may lead to fibrogenesis [136]. Lu et al. identified that AN modulates a signaling cascade that induces HIF-1α expression in OC cells [116]. The eventual initiation of autophagy was helpful to cell survival from AN-induced apoptosis [116]. Additionally, chronic stimulations of AN improve OC and leukemia T cells’ tolerance to anti-cancer medications, as well as to hypoxia and glucose deprivation, and increase autophagy activity, which increases drug resistance [137].

**Figure 5:**
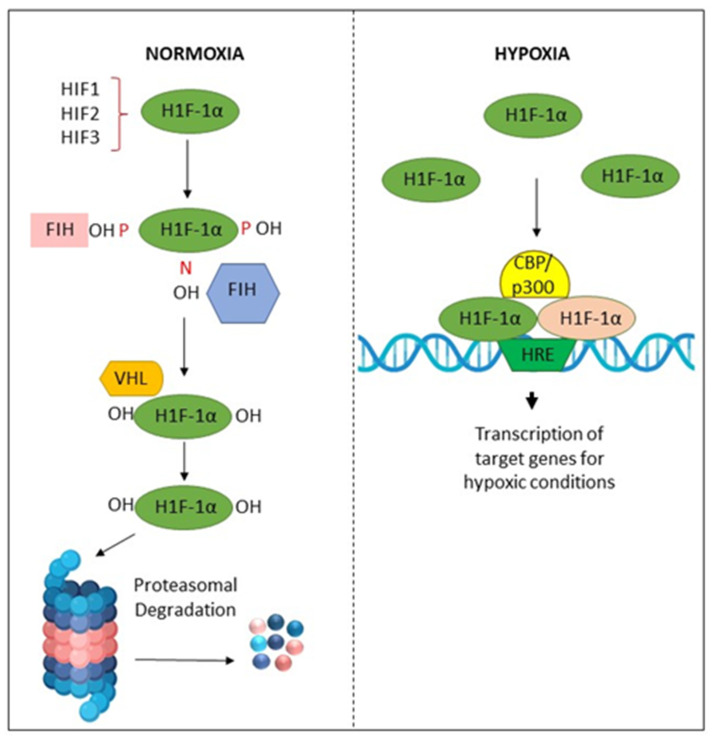
Activation and degradation of the hypoxia-inducible factor-1α (HIF-1α). In normoxia, HIF-1α is degraded rapidly, while in hypoxic conditions, it is accumulated. HIF-1α associates with HIF-1β, and the resulting heterodimer binds to the hypoxia response element (HRE) of target genes. The factor inhibiting HIF-1 (FIH-1) is a protein that binds to HIF-1α and inhibits its transactivation function. The von Hippel–Lindau (VHL) protein is a tumor suppressor.

Tissue hypoxia may also promote EMT by activating several transcriptional factors, including Twist1 and Snail, to promote the growth of tumors [138,139]. In addition, previous studies have shown the association between HIF-1α and vascular endothelial growth factor (VEGF) in OSCC, and elevated levels of HIF-1α expression appear to predict a poor prognosis. Prolyl hydroxylases (PHDs) modify HIF-1α and prepare it for proteasomal degradation at physiological concentrations of O_2_. In hypoxic conditions, these PHDs are inhibited, and HIF-1α dimerizes with HIF-1β to form HIF-1, which is responsible for the activation of several genes, including VEGF, which is an essential regulatory gene of angiogenesis in the adaptation to a hypoxic microenvironment [140]. Hypoxic conditions may also promote resistance in the tumor microenvironment by activating pathways linked to stemness, such as Sox2, AKT/Notch1, and Oct3/4 molecular signals [141,142] (Figure 5).

### 2.9. Areca Nut Metabolites on the Oral Microbiome

The oral microbiome is the collection of microorganisms that live in the mouth, including bacteria, viruses, and fungi [143]. A healthy oral microbiome is essential for maintaining oral health and preventing disease, including oral cancer [144]. 

Researchers investigate the potential impact of Areca nut on the oral microbiome. Several studies have found that Areca nut metabolites, specifically arecoline and arecaidine, can alter the composition and diversity of the oral microbiome [145]. These changes can lead to a reduction in beneficial bacteria and an increase in harmful bacteria, which can cause oral inflammation and oxidative stress. Inflammation and oxidative stress are known risk factors for oral cancer [146].

A study by Chen et al. (2022) found that Areca nut metabolites altered the composition and diversity of the oral microbiome, leading to a reduction in beneficial bacteria, such as *Lactobacillus*, and an increase in harmful bacteria, such as *Porphyromonas gingivalis* [147]. These changes can cause oral inflammation and oxidative stress, increasing the risk of oral cancer [145]. Furthermore, Hernandez et al. (2017) found that Areca nut use led to changes in the oral microbiome, including an increase in the number of potential carcinogenic bacteria, such as *Fusobacterium nucleatum* [148,149], and a reduction in the number of beneficial bacteria, such as *Streptococcus salivarius* [149]. This shift in the oral microbiome can increase the risk of oral cancer by promoting inflammation and oxidative stress [148].

In a systematic review and meta-analysis of the impact of Areca nut on the oral microbiome, Zhong et al. (2021) found that Areca nut use led to significant alterations in the oral microbiome, including changes in the abundance and diversity of bacterial species. These changes may increase the risk of oral cancer by promoting inflammation and oxidative stress [149].

## 3. Conclusions

AN is an addictive substance widely consumed by all age groups, specifically in Southeast Asia. Apart from being a carcinogenic agent, it may have negative effects on the human body, impacting nearly all organs. Numerous in vitro and in vivo investigations have demonstrated AN’s carcinogenicity, mutagenicity, and genotoxicity, evidencing its position as a carcinogen beyond doubt. In addition, AN metabolites can significantly impact the oral microbiome, leading to changes that may increase the risk of oral carcinogenesis. Many molecules involved in cell cycle control, DNA damage, hypoxia, cell senescence, and many other biological processes related to carcinogenesis were studied, and there is substantial evidence for AN-induced malignant transformation in OSF. 

Furthermore, OSF incidence is high in geographical regions where habitual chewing of AN is also prevalent. Comparatively, OC patients with a frequent AN chewing habit were exposed to more aggressive cancer phenotypes, with elevated rates of cancer metastasis, recurrence, and poor patient survival. Hence, the evidence points to the conclusion that ANs lead to oral carcinogenesis via complex mechanisms. It is evident that harmful and addictive substances in AN affect the whole human body, and its consumption is essential to be regulated for the well-being of society.

## Figures and Tables

**Table 1:** Summary of Key Literature.

Nature of Extract(s)	Type of Experiment	Analyses Conducted	Main Observations	Reference
Aqueous AN and pan masala extracts	Injection into buccal mucosa of Sprague-Dawley rats	Histological analysis and TGF-beta1 gene by RT-PCR	Epithelial atrophy and collagen accumulation, significant upregulation of TGF beta1 gene	[13]
AN extract	Subcutaneous injection into BALB/C mice	Histological analysis, immunohistochemical staining, and immunoblotting	Increase of collagen deposition, higher expression of α-smooth muscle actin, and connective tissue growth factors compared to control group	[14]
Arecoline	Smearing in the inner mouth area of C57BL/6 mice followed by administration via drinking	Examination of tongue tissue, Krt17 protein expression analysis	Malignant lesions observed, and upregulation of Krt17 compared to control group	[15]
Arecoline	In vitro exposure of arecoline on human gingival fibroblasts	Analysis of cytotoxicity, mitochondrial activity, and cell cycle analysis	DNA inhibition, decrease of mitochondrial activity, and cell cycle arrest at the G2/M phase in a dose-dependent manner	[17]
AN extract and arecoline	In vitro exposure on human gingival tissue	Cytotoxicity, total and unscheduled DNA synthesis	AN extract caused cell growth suppression, and induction of total and unscheduled DNA synthesis at lower concentrations than arecoline	[18]
Aqueous AN and aqueous arecoline extracts	In vitro exposure on mouse kidney cells	Cell growth and DNA strand break analysis	Suppression of cell growth and enhanced DNA strand breaks caused by exposure to AN or arecoline compared to control group	[20]
Aqueous, acetic acid, hydrochloric acid, and ethanol extracts of AN	In vitro treatment on Hep 2 cells	Cell viability and unscheduled DNA synthesis	Reduction of cell viability and increase of unscheduled DNA synthesis observed, with aqueous and acetic acid extracts showing a higher effect than other extracts	[21]
AN extract	In vitro treatment on normal human oral keratinocytes	Cell viability and proliferation, p38MAPK and repair enzymes, cell cycle, NF-κB, and IκBα activation	Inhibition of cell viability and proliferation, p38MAPK activation, cell cycle arrest at G1 phase, induction of NF-κB and IκBα	[22]
Aqueous AN extract	In vitro treatment on Chinese hamster ovary cells	Cytotoxicity, intracellular ROS production and micronuclei formation, cell cycle analysis, evaluation of actin filament distribution and nucleus number	Increased MN frequency, G2/M arrest, cytokinesis failure, and accumulation of hyperploid/aneuploid cells increased intracellular H_2_O_2_ levels and actin filament disorganization	[23]
Arecoline extracts	In vitro exposure of arecoline on human gingival fibroblasts	Cytotoxicity assay and gene expression profiling	Increased cytotoxicity in a dose-dependent manner, the genes AKR1A1, CYP26B1, S100A12, ALDH9A1, MAOA, UGCGL1, and GSS, LCMT1, and NAT8 were all repressed by arecoline. Gene related to DNA damage signaling (DDIT4) was moderately induced. DNA repair-related genes BRCA1 repressed, and RAD50 were induced by arecoline	[24]
AN extract, arecoline and arecaidine	In vitro exposure of AN extract on human keratinocytes	Cytotoxicity assay, apoptosis, ROS analysis, and hypoxanthine phosphoribosyltransferase (HPRT) mutation.	Increased HPRT mutations, intracellular ROS generation, and apoptosis	[25]
AN extract	In vitro exposure of AN extract on human gingival fibroblasts	Cytokinin secretion, ROS production, oxidative DNA damage, DNA double-strand breaks, gene silencing	GRO-α, IL-6, and IL-8 cytokinin production was enhanced. Results indicate NOX1 and NOX4 gene-mediated cytokine-induced oxidative DNA damage by regulating ROS production	[26]
AN extract and arecoline	In vitro exposure of AN extract and arecoline on human keratinocytes compared to KB carcinoma cells	mRNA expression, extracellular signal-regulated kinase (ERK) phosphorylation via RT-PCR, flow cytometry, Western blotting, and ELISA	Induced c-Fos mRNA expression and PGE2 and IL-6 production by cells and stimulation of ERK-1/ERK2 phosphorylation	[27]
AN extract	In vitro exposure of AN extract on human gingival keratinocytes	Cytotoxicity, mRNA and protein expression, and ELISA	Extract stimulated PGE2/PGF2α production, and upregulated expression of cyclooxygenase-2 (COX-2), cytochrome P450 1A1 (CYP1A1) and hemeoxygenase-1 (HO-1)	[28]
Arecoline	In vitro exposure of arecoline extract on human buccal mucosal fibroblasts	Gene expression, collagen contraction, and migration capability	Increased Twist expression transcript and protein levels; myofibroblast activity, including collagen gel contraction and migration capability	[29]
AN extract	In vitro exposure of AN extract on human gingival fibroblasts and epithelial cells compared to TGF-β treatment	Transcriptome profiling	AN and TGF-β enhanced fibroblast activation in both types of cells. Both significantly common and unique gene expression patterns were identified in both types of cells. Action of AN on fibroblasts is enhanced by epithelial-mesenchymal interaction via TGF-β	[30]
Arecaidine	Intraperitoneal injection into Swiss albino mice	Sister chromatid exchange analysis	Sister chromatid exchange frequency increased dose-dependently	[31]
Arecoline	Organ-specific mutagenic potential in gpt delta transgenic mice	Genomic DNA analysis from the oral tissues and liver tissues	G:C to T:A transversions (in oral tissues) and G:C to A:T transitions (in oral tissues and liver tissues) were observed	[32]
Arecoline	Cytotoxic and genotoxic effects of arecoline in normal rat hepatocytes	Cell cycle analysis, DNA damage, TGF-β1 mRNA expression, protein expression, phosphorylation of p53	Arecoline induces cell cycle arrest, and DNA damage, increasing TGF-β1 mRNA expression and transcription. Also, arecoline increased p21WAF1 protein expression and p53 phosphorylation and gene transcription	[33]

## Data Availability

Not applicable.
